# Selection on multiple sexual signals in two Central and Eastern European populations of the barn swallow

**DOI:** 10.1002/ece3.5629

**Published:** 2019-09-04

**Authors:** Péter L. Pap, Attila Fülöp, Marie Adamkova, Jaroslav Cepak, Romana Michalkova, Rebecca J. Safran, Alexandru N. Stermin, Oldrich Tomasek, Csongor I. Vágási, Orsolya Vincze, Matthew R. Wilkins, Tomas Albrecht

**Affiliations:** ^1^ Evolutionary Ecology Group Hungarian Department of Biology and Ecology Babeş‐Bolyai University Cluj Napoca Romania; ^2^ MTA‐DE Behavioural Ecology Research Group Department of Evolutionary Zoology and Human Biology University of Debrecen Debrecen Hungary; ^3^ Department of Ecology and Evolutionary Biology University of Colorado Boulder CO USA; ^4^ Institute of Vertebrate Biology Czech Academy of Sciences Brno Czech Republic; ^5^ Department of Botany and Zoology Masaryk University Brno Czech Republic; ^6^ Bird Ringing Station National Museum Praha Czech Republic; ^7^ Department of Zoology Faculty of Science Charles University Prague Czech Republic; ^8^ Department of Taxonomy and Ecology Babeş‐Bolyai University Cluj Napoca Romania; ^9^ Center for Science Outreach Vanderbilt University Nashville TN USA

**Keywords:** barn swallow, *Hirundo rustica rustica*, sexual selection, tail length, ventral coloration

## Abstract

Variation in intensity and targets of sexual selection on multiple traits has been suggested to play a major role in promoting phenotypic differentiation between populations, although the divergence in selection may depend on year, local conditions or age. In this study, we quantified sexual selection for two putative sexual signals across two Central and East European barn swallow (*Hirundo rustica rustica*) populations from Czech Republic and Romania over multiple years. We then related these differences in selection to variation in sexual characters among barn swallow populations. Our results show that tail length and ventral coloration vary between populations, sexes, and age classes (first‐time breeders vs. experienced birds). We found that selection on tail length was stronger in first‐time breeders than in experienced birds and in males than in females in the Romanian population, while these differences between age groups and sexes were weak in Czech birds. We suggest that the populational difference in selection on tail length might be related to the differences in breeding conditions. Our results show that ventral coloration is darker (i.e., has lower brightness) in the Romanian than in the Czech population, and in experienced birds and males compared with first‐time breeders and females, respectively. The sexual difference in ventral coloration may suggest sexual selection on this trait, which is supported by the significant directional selection of ventral coloration in first‐time breeding males on laying date. However, after controlling for the confounding effect of wing length and tarsus length, the partial directional selection gradient on this trait turned nonsignificant, suggesting that the advantage of dark ventral coloration in early breeding birds is determined by the correlated traits of body size. These findings show that ventral coloration may be advantageous over the breeding season, but the underlying mechanism of this relationship is not clarified.

## Graphical abstract

In this study, we quantified sexual selection for two putative sexual signals across two Central and East European barn swallow (*Hirundo rustica rustica*) populations from Czech Republic and Romania over multiple years. We suggest that the populational difference in selection on tail length might be related to the differences in breeding conditions for first‐time breeders and experienced birds. Our results show that ventral colouration may be advantageous over the breeding season, but the underlying mechanism of this relationship is not clarified.

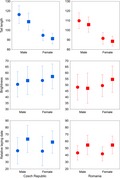

## 1 INTRODUCTION

Variation in sexually selected traits—traits used in mate selection—have been suggested to play a major role in promoting the phenotypic differentiation between populations, which may ultimately lead to morphological diversity, reproductive isolation during population divergence, and species formation (Boul, Chris Funk, Darst, Cannatella, & Ryan, 2007; Coyne & Orr, 2004; Saetre et al., 1997). Divergence in sexual signals due to differential targets of female preference may explain apparent morphological differences among recently diverged populations and subspecies, and population structure may eventually evolve if divergent signals lead to assortative mating (van Doorn, Edelaar, & Weissing, 2009; Vortman, Lotem, Dor, Lovette, & Safran, 2013). A first step in understanding whether secondary sexual traits differ between populations as a function of divergent mate preference entails careful, longitudinal studies of how phenotype variation is associated with reproductive performance.

Multiple male ornamental traits typically co‐occur and are presented to choosy females simultaneously, with expression and signaling information depending on their production and maintenance costs (Bro‐Jørgensen, 2010). The divergence between populations in sexual signals can be related to the difference between signals in their production costs and sensitivity to environmental conditions (Candolin, 2003; Maan & Seehausen, 2011). Therefore, variation in sexual traits between populations may depend on year, because climatic conditions affect the expression of sexual traits and geographically distinct populations experience differences in conditions between years (Chaine & Lyon, 2008; Reudink et al., 2015; Saino et al., 2004). Thus, the reported variation in phenotypes between populations may depend on the scale and intensity of the study.

One classical example of sexual selection and the role of secondary sexual traits in phenotypic differentiation in the animal kingdom is the broadly distributed barn swallow, *Hirundo rustica*, which has six distinctive subspecies (Møller, 1994; Scordato & Safran, 2014). All subspecies are fairly similar in their ecological niche as they are aerial feeders and, except for two middle‐eastern distributed subspecies, are highly migratory (Turner, 2006). Phenotypic differentiation between these subspecies is associated with divergent sexual selection among barn swallow subspecies (Safran et al., 2016; Scordato & Safran, 2014; Vortman et al., 2013). Moreover, the direction of phenotypic divergence is consistent with differences in sexual selection pressures among subspecies (Vortman et al., 2013; Wilkins et al., 2016). For example, the Israeli *H. r. transitiva* subspecies, which is characterized with dark ventral plumage, experiences directional selection for darker plumage. Similarly, the European *H. r. rustica*, which has the longest tail feathers of any subspecies, experiences directional selection for elongated tail feathers (Møller et al., 2006; Wilkins et al., 2016). In completion of this and other observational studies (for review see Scordato and Safran (2014)), experimental results show that dark coloration in North American and Israeli populations and the longer streamer length in Israeli populations are associated with increased reproductive outcomes (Safran et al., 2016), indicating that sexual selection favors different combinations of the same traits in recently diverged, yet geographically isolated populations of barn swallows.

One of six well‐characterized subspecies within the larger *H. rustica* complex, the European barn swallow, *H. r. rustica*, has been the subject of intense research over the last thirty years, with an emphasis on sexual selection (Romano, Costanzo, Rubolini, Saino, & Møller, 2017). Previous research across 22 European and North African populations of *H. r. rustica*, not sampled here, demonstrated consistent directional selection for elongated tail feathers, as the intensity of selection with respect to breeding date (a measure of fitness) was significantly correlated for tail length across populations (Møller et al., 2006). This work suggests that females mate preferentially with long‐tailed males; however, the strength of selection on tail length differed significantly among populations. Importantly, geographical patterns of phenotypic selection predict current patterns of phenotypic variation among populations (Møller, 1994). However, a recent study on two Central and Eastern European populations of *H. r. rustica* from the Czech Republic (the same populations sampled here) and Turkey shows that the selection for long tail in male barn swallows is weak (Wilkins et al., 2016), suggesting a relatively reduced function of this trait in mate choice compared to other populations from Europe (e.g., Denmark, Italy, and Spain; (Romano et al., 2017). Albeit, the intensity of selection on sexual traits may be underestimated due to the confounding effect of age, since it appears that the selection on at least one trait, the tail length, differs between barn swallows breeding for the first time and experienced birds (Møller et al., 2006). The geographical variation in selection strength indicates that the function of the tail length in mate choice decreases in populations toward lower latitudes situated in the proximity of *H. r. transitiva* subspecies found in Israel (Møller et al., 2006; Wilkins et al., 2016). Barn swallows belonging to *H. r. transitiva* are characterized by a different set of sexual characters (darker ventral coloration and shorter tail), and because of the absence of physical barriers between the two subspecies, admixture between the two may explain the displacement in sexual traits and reduced selection on tail length in Central and East European birds compared with westerly populations (Wilkins et al., 2016).

Besides the long tail, recent studies suggest that ventral coloration can be important in mate choice and reproductive isolation in the European population of *H. r. rustica*. A study on an Italian population suggests sexual dimorphism in coloration of ventral feathers (Parolini et al., 2017; Saino, Romano, Rubolini, Teplitsky, et al., 2013), though the intensity of coloration is smaller than in *H. r. transitiva* (Wilkins et al., 2016). On the same Italian population, darker males had significantly higher seasonal reproductive success (Parolini et al., 2017), and survival was higher in males with relatively higher eumelanin‐to‐pheomelanin ratio of ventral body feathers (Saino, Romano, Rubolini, Ambrosini, et al., 2013). However, ventral coloration did not predict the lifetime reproductive success of these birds (Costanzo, Ambrosini, Caprioli, Gatti, Parolini, Canova et al., 2017). It appears that sexual selection acts on the ventral coloration of females as well, because females with higher UV reflectance of their ventral plumage coloration were more promiscuous and the proportion of male offspring in a nest increased with maternal plumage darkness (Costanzo, Ambrosini, Caprioli, Gatti, Parolini, Romano et al., 2017; Romano et al., 2015). Contrary to these findings, the study on Czech and Turkish populations of *H. r. rustica* shows that the selection for dark ventral coloration in male barn swallows is weak (Wilkins et al., 2016), suggesting a reduced function of this trait in mate choice compared to the Italian population. Taken together, it is clear that the selection for large and colorful traits and their advantage in mate choice differs between populations. Therefore, studies that enable direct comparisons with other populations, which are possibly exposed to different sexual selection, will fill this gap in our knowledge about the potential role of sexual signals in morphological differentiation and reproductive isolation both within and among the subspecies of barn swallow.

In this study, we utilize two long‐term datasets from two Central and Eastern European populations, which are closely located to the *H. r. transitiva* subspecies. Birds from these populations preferentially use the East‐European migratory flyway (Klvaňa et al., 2018), which may possibly affect the magnitude of admixture with *H. r. transitiva* and separate from more westerly and southerly *H. r. rustica*. We use the data collected from these two populations to investigate the relative importance of sexual selection in phenotypic expression of two putative secondary sexual traits. Collectively, 1,204 measures were taken from 828 individuals over seven and eight years, respectively, from the Czech Republic (Central Europe) and Romania (Eastern Europe). We estimate selection differentials and partial selection differentials (controlling for body size) on laying date and secondary sexual traits of male and female barn swallows that have been shown to have a role in sexual selection in one or more of the geographical populations/subspecies of this species (Romano et al., 2017; Scordato & Safran, 2014). Specifically, using laying date as an estimate of fitness (Møller et al., 2006) we calculated selection on the length of the outermost tail feathers and on melanin‐based coloration of the white to chestnut ventral plumage region of male and female barn swallows. We calculated selection on these phenotypic traits for both sexes to give an estimate for the difference between sexes, which can be a proxy of the strength of sexual selection. Further, because first‐breeders and experienced birds differ in arrival date and consequently in their access to breeding sites (Møller, 1994), the strength of sexual selection may change with age. Therefore, we measured the strength of selection on multiple sexual traits separately for first‐breeders and experienced barn swallows.

## 2 MATERIAL AND METHODS

### 2.1 Field methods

We studied barn swallows breeding at two populations located within the Třeboňsko Protected Landscape Area in South Bohemia (Doudlebia, Czech Republic) and in Cojocna village (central Transylvania, Romania) over eight (2010–2017) and seven years (2011–2017), respectively. Nests from the Czech population were located at two isolated farms (Šaloun, Lomnice nad Lužnicí and Hamr, Lužnice; Petrželková et al., 2015) where barn swallows breed in colonies of up to 40 pairs. Birds from Romania were located in stall buildings where they breed mostly solitarily or in small colonies of up to ten pairs (Fülöp, Vágási, & Pap, 2017). The number of nests we monitored in both populations varied between 50 and 120 each year. Barn swallows were systematically captured using mist nets or nest‐traps over the course of the breeding season, and each individual was tagged with an aluminium ring (both populations) and marked with unique color combination of 1–2 colored plastic rings (Czech population), which allowed us to identify the position of their nest. Standard morphological measurements were taken from all individuals in all capture years, including length of both outermost tail feathers, wing and tarsus length. Tail length was expressed as the mean of the left and right, unbroken streamer. At least ten contour feathers were collected from the ventral region for later spectrometric color measurements (see below) from adult birds captured over the whole study period in the case of the Czech population, and in 2017 from the Romanian population. Sex was determined by visual examination of the presence of a brood patch (only females develop a brood patch) and for each nest, we examined nests every 4–5 days to determine the laying date (appearance of the first egg), clutch size, and brood size at days 1 and 10 to 12 posthatching. Barn swallow populations from both countries have been carefully monitored in a series of studies, with more than 95% of all breeding birds captured annually. Previous observations of barn swallows (Schaub & von Hirschheydt, 2009), and our own capture—recapture data, indicate high breeding fidelity for adults (Pap, Tökölyi, & Szép, 2005; Pap et al., 2018; Petrželková et al., 2015). Because of the high breeding site fidelity and constant ringing effort, individuals that were captured in any year between 2011 and 2017 for the Czech and 2012 and 2017 for the Romanian populations, and had not been captured as adults in the previous year, could be assumed to be 1‐year‐old individuals at their first breeding season. Immigrating from colonies outsides our study area was rare, except in rare cases when they were local recruits (i.e., individuals that were ringed as nestlings at the studied population, allowing us to directly determine their age). We could thus categorize age in all breeding birds as 1‐year‐old (hereafter first‐time breeders) or more than 1‐year‐old (hereafter experienced birds) except for the first study year, when aging was not possible. Therefore, the data from the first study year (2010 for Czech and 2011 for the Romanian population) were excluded from all subsequent analyses.

### 2.2 Feather measurements

Samples of plumage were removed by gently plucking at least ten feathers from the ventral body region. Ten feathers were arranged together and fixed on a white paper index cards to achieve a layer equivalent to real ordering of feathers on the body of the bird. The reflectance of these feathers was measured with a spectrometer, model AvaSpec 2048 (Avantes, Netherlands), and an AvaLight‐XE (Avantes, Netherlands) was used as a light source. The sensing head of the spectrometer was modified by using of self‐made adapter that secure standardized light conditions by screening out the ambient light and maintains a constant distance of 3.5 mm between sensing head and measured sample. Each sample was measured three times at the distal part of the feather under an angle of 90°. The spectrometer was regularly calibrated after measurement of samples of each two individuals using WS‐2 (Avantes, Netherlands) white color standard and absolute dark. Measurements were converted using the software program AvaSoft 7.8. Spectral data were analyzed in R 3.4.3 (R Development Core Team, 2017) using the R package pavo version 1.3.1 (Maia, Eliason, Bitton, Doucet, & Shawkey, 2013). Spectral data were trimmed to the range of wavelengths between 300 and 700 nanometers. Reflectance equivalent to wavelengths in integral numbers was established. Three measurements of each sample were averaged, and this average was smoothed by a span of 0.2. We calculated hue, chroma, and brightness for objective quantification of color variation. We used only brightness as the main color metric, because all three color metrics were previously found to be highly intercorrelated across the breast and belly of individual barn swallows (McGraw, Safran, & Wakamatsu, 2005; Safran & McGraw, 2004), and brightness is the most variable among‐individual dimension of color in this body region. Lower brightness scores (% reflectance) indicate plumage color that appears darker, redder, and more saturated when compared to feathers with higher brightness scores, as shows a study on North American barn swallows (Safran & McGraw, 2004).

### 2.3 Statistical analyses

We tested whether phenotypic traits and laying date differed among populations, sexes and age categories by building separate linear models (LMs) and linear mixed‐effects models (LMEs; Bates, Mächler, Bolker, & Walker, 2015) for each of these parameters, including population, sex, age, year as fixed factors, and the identity of birds as a random factor. We report minimal models, in which all main effects and significant interactions were retained; these were all obtained using stepwise backward elimination based on the largest nonsignificant (>.05) *p* value.

We then calculated standardized directional (*s*) and quadratic (*g*) selection differentials and partial selection differentials (i.e., directional *β* and quadratic *γ* gradients, controlling for body size [see below]) in separate LMs and LMEs for each secondary sexual trait (tail length, ventral coloration), for each population, age group, and sex, following Lande and Arnold (1983) and (Brodie, Moore, & Janzen, 1995). Selection coefficients (differentials and gradients) provide an estimate of the change in phenotype in standard deviation units for a unit change in fitness. All phenotypic traits were scaled and centered by subtracting from each population mean and dividing by the standard deviation. Relative fitness was calculated by dividing individual values by the mean fitness for each population and used as the response variable.

Selection differentials were estimated as the regression coefficient of relative fitness on standardized phenotype, as described in Lande and Arnold (1983). Partial selection differentials (gradients), controlling for indirect selection on a trait due to selection on other traits (here wing and tarsus length) that are correlated with the secondary sexual trait in question, were estimated using multiple linear regression with the standardized fitness component (clutch initiation date) as the dependent variable and the standardized phenotypic characters as independent variables (Lande & Arnold, 1983). We decided to include both wing and tarsus length in these models, as they may reflect distinct allometric impacts of body size on the expression of secondary sexual traits (all correlations between tarsus and wing length, populations and sexes analyzed separately, Pearson's correlation, *p* > .110). We calculated differentials and gradients for the whole dataset including all years and in these models, the identity of individuals was included as a random factor, except for the brightness of the Romanian birds for which we had data only for one year and first‐time breeders.

For tail streamers, the trait for which we had measurements across multiple breeding seasons, we also calculated selection coefficients separately for each year and these values were used in a GLM to analyse differences in selection between populations, sexes, and age. Quadratic selection estimates were calculated as the coefficients of the second‐order polynomial term in models where secondary sexual signaling traits were included as explanatory variables separately. In order to insure the computation of unbiased selection coefficients based on polynomial effect sizes, quadratic estimates, and standard errors were doubled, following Stinchcombe, Agrawal, Hohenlohe, Arnold, and Blows (2008).

Our measure of fitness was clutch initiation date, as this fitness component correlates strongly with fledging success (Møller, 1994) and the effect size (the relationship between laying date and the expression of secondary sexual traits), a measure of the intensity of sexual selection, is large (Romano et al., 2017). Moreover, in a previous study, sexual selection differentials calculated from female clutch initiation and fledging success were strongly correlated across 22 European and North African populations (Møller et al., 2006), reinforcing the suitability of this reproductive metric as a surrogate of fitness. For interpreting significance of selection coefficients, we corrected *P*‐values for false discovery rate, as this is a superior method for controlling analysis‐wide type I error when performing multiple comparisons (Benjamini, Drai, Elmer, Kafkafi, & Golani, 2001; Nakagawa, 2004). All statistical analyses were conducted in the R statistical environment, version R 3.5.3 (R Development Core Team, 2017).

## 3 RESULTS

We tested the covariation between two putative secondary sexual traits separately for the Czech and Romanian populations and for males and females in linear and mixed‐effect models, where age (fixed effect), identity of individuals and year (random effects) were controlled for. Tail length was not predicted by brightness in any of the populations and sexes (*χ* ^2^ < 3.54, *N* > 289, *p* > .060), which indicates that these traits may show different aspects of signaling in this species.

**Figure 1:**
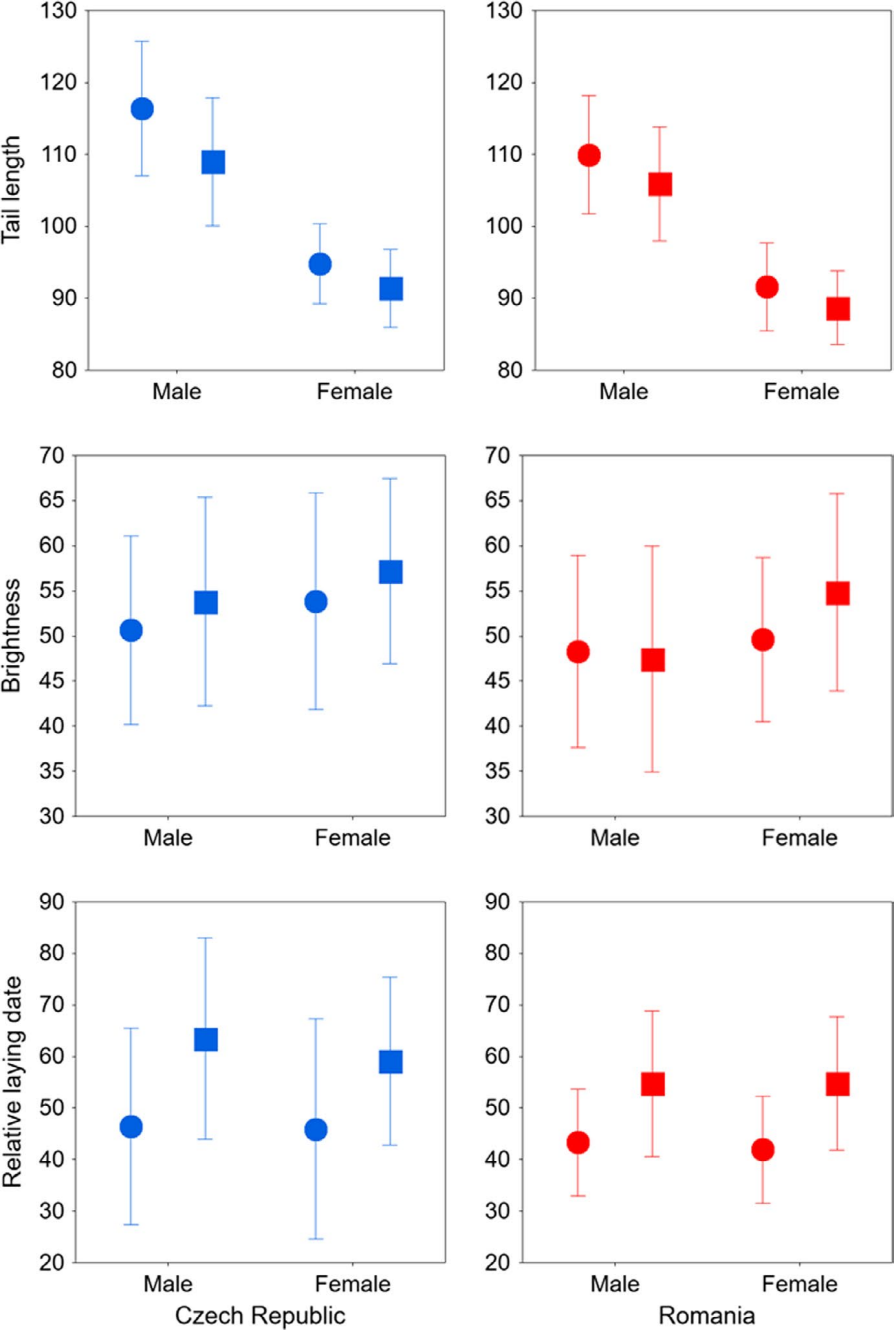
Representation of the variation in the two putative sexual traits and the laying date measured in experienced (circle) and first‐time breeders (square) male and female barn swallows from the Czech (blue) and Romanian (red) populations. Mean ± *SD*

Table 1 and Figure 1 demonstrate variation in two putative secondary sexual traits and in the laying date. Tail length was significantly higher in the Czech than in the Romanian population, and in experienced birds and males compared with first‐time breeders and females, respectively. The significant age and sex interaction show that the increase in tail length with age was higher in males than in females. Brightness was lower in the Romanian than in the Czech population (darker color in the former), and in experienced birds and males compared with first‐time breeders and females, respectively. Differences between age groups and sexes were similar between populations, as indicated by the absence of significant interaction between main factors. Barn swallows started to lay earlier in the Romanian than in the Czech population, and experienced birds bred earlier than yearlings in both females than males.

**Table 1:** Variation in phenotypic secondary sexual traits and laying date between populations, sexes, age classes (first‐time breeders and experienced birds), and study years of barn swallows from Czech and Romanian populations. Results are from minimal fixed effect models. Significant effects are marked in bold

	*df*	*χ^2^*	*p*
Tail length (*N* = 828 individuals)			
Population	1	30.12	**.0001**
Age	1	126.51	**<.0001**
Sex	1	1,085.33	**<.0001**
Population × Age	1	5.13	**.024**
Age × Sex	1	9.39	**.002**
Ventral feather brightness (*N* = 505 individuals)			
Population	1	9.62	**.002**
Age	1	4.04	**.044**
Sex	1	16.67	**<.0001**
Year	6	157.39	**<.0001**
Laying date (*N* = 828 individuals)			
Population	1	9.13	**.003**
Age	1	5.93	**.015**
Sex	1	4.88	**.027**
Year	6	9.97	.126
Age × Year	6	50.08	**<.0001**

Standardized directional selection differentials were significant or marginally significant for the tail length of the first‐time breeding males from the Romanian and Czech populations, respectively (Table 2a). Similarly, brightness of the ventral feathers of the first‐time breeding males from both populations was under significant directional selection, showing that barn swallows with long tails and dark ventral coloration started to breed earlier. Selection differentials on secondary sexual traits of experienced males from both populations were nonsignificant. Controlling for the correlative effect of wing length and tarsus length on secondary sexual traits proved that only the tail length of first‐time breeding males from the Romanian population is under significant directional selection (Table 2b), after correction of the significance values for false discovery rate.

**Table 2:** Standardized directional selection differentials and gradients (controlling for the effect of wing length and tarsus length) for the two putative sexual traits measured in first‐time breeders and experienced male and female barn swallows from Czech (CZ) and Romanian (RO) populations. Significant effects (in case of partial directional selection differential after correcting for false discovery rate) are marked in bold

(A) Standardized directional selection differentials								
	CZ				RO			
	*N*	*s* (*SE)*	*t*	*p*	*N*	*s* (*SE*)	*t*	*p*
Male								
Tail length								
First‐time breeders	134	–0.043 (0.022)	–1.96	.0520	**138**	**–0.065 (0.021)**	**–3.11**	**.0023**
Experienced birds	156	–0.015 (0.030)	–0.49	.6268	159	–0.004 (0.017)	–0.21	.8349
Ventral feather brightness								
First‐time breeders	**134**	**0.051 (0.022)**	**2.36**	**.0200**	**17**	**0.099 (0.042)**	**2.38**	**.0310**
Experienced birds	156	–0.051 (0.029)	–1.75	.0819	17	0.050 (0.048)	–1.03	.3204
Female								
Tail length								
First‐time breeders	161	–0.023 (0.018)	–1.28	.2039	178	–0.019 (0.018)	–1.06	.2898
Experienced birds	**150**	**–0.098 (0.034)**	**–2.93**	**.0041**	128	0.024 (0.022)	1.10	.2718
Ventral feather brightness								
First‐time breeders	161	0.015 (0.018)	0.86	.3934	**14**	**–0.076 (0.033)**	**–2.29**	**.0410**
Experienced birds	150	–0.015 (0.034)	–0.45	.6520	16	–0.048 (0.034)	–1.39	.1857

(B) Partial directional selection differentials, controlling for wing and tarsus length										
	CZ					RO				
	*N*	*β* (*SE*)	*t*	*p*	*p adj*	*N*	*β* (*SE*)	*t*	*p*	*p adj*
Male										
Tail length										
First‐time breeders	134	–0.038 (0.023)	–1.67	.0978	.1572	**138**	**–0.078 (0.023)**	**–3.43**	**.0008**	**.0024**
Experienced birds	156	–0.020 (0.033)	–0.62	.5397	.8096	159	0.001 (0.019)	0.04	.9666	.9666
Ventral feather brightness										
First‐time breeders	134	0.050 (0.021)	2.33	.0213	.0639	17	0.099 (0.046)	2.17	.0493	.1480
Experienced birds	156	–0.050 (0.030)	–1.69	.0926	.2779	17	–0.050 (0.049)	–1.02	.3245	.4868
Female										
Tail length										
First‐time breeders	161	–0.028 (0.019)	–1.47	.1444	.4053	178	–0.018 (0.019)	–0.94	.3497	.8480
Experienced birds	**150**	**–0.114 (0.038)**	**–2.98**	**.0035**	**.0104**	128	0.017 (0.023)	0.75	.4543	.6020
Ventral feather brightness										
First‐time breeders	161	0.015 (0.018)	0.83	.4104	.6153	14	–0.071 (0.038)	–1.85	.0949	.2846
Experienced birds	150	–0.022 (0.035)	–0.63	.5316	.7975	16	–0.049 (0.046)	–1.07	.3054	.8996

Standardized directional selection differentials were significant for the tail length of experienced female barn swallows from the Czech population and for the brightness of first‐time breeding females from the Romanian population, respectively (birds with long tails and light ventral coloration bred earlier; Table 2a). After controlling for the correlative effect of wing length and tarsus length on secondary sexual traits, only the selection gradient on the tail length of experienced females from the Czech population remained significant (Table 2b).

**Figure 2:**
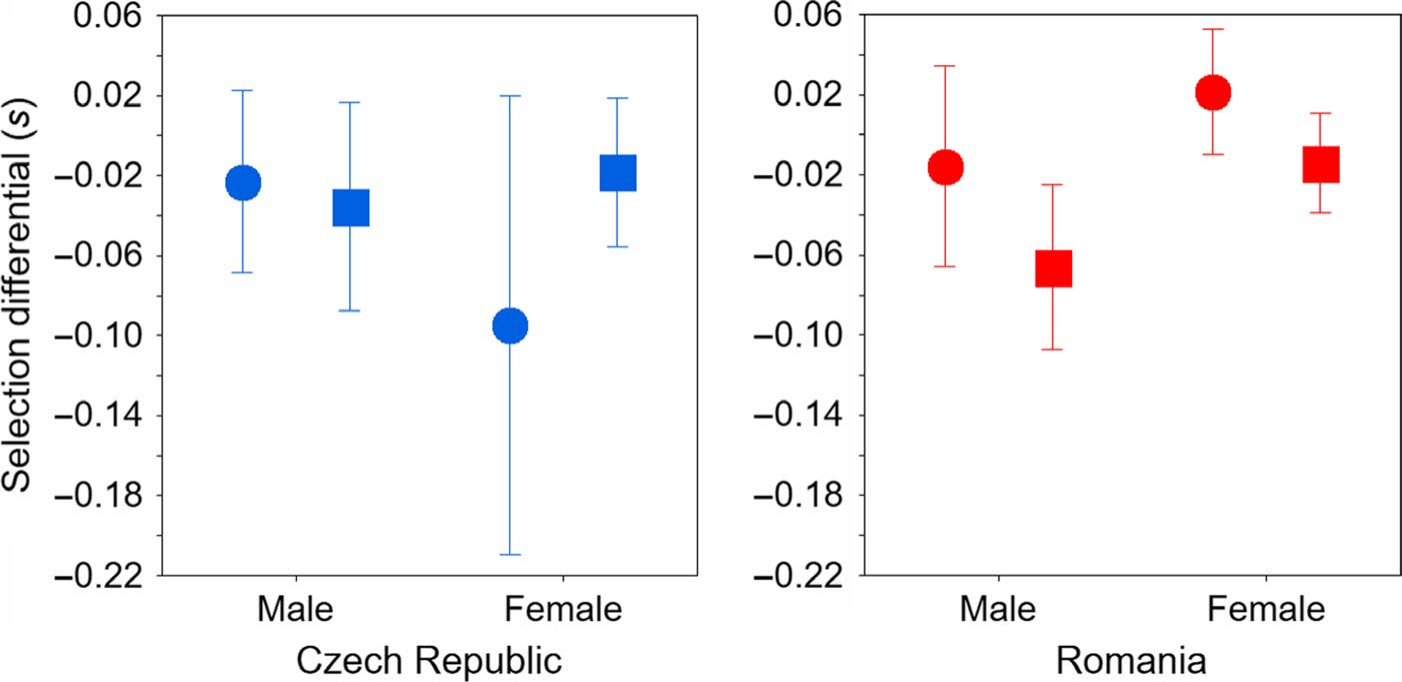
Difference between sexes and age categories (circle—experienced birds, square—first‐time breeders) in the selection differential for the tail length measured over subsequent years in the Czech (2011–2017) and the Romanian (2012–2107) barn swallow population (for the statistics see Table 3). Mean ± *SD*

The GLM of the difference between sexes, age categories, and populations in the standardized selection differential for the tail length shows that in the Czech population, selection on this trait is similar between age groups and between males and females (Table 3a, Figure 2). In the Romanian population, however, the selection differential on tail length was higher for the first‐time breeders than experienced birds and for males than for females, respectively. When the selection differentials of the two populations were analyzed together, the significant population × age and population × sex interactions revealed differences between age groups and sexes among populations. That is, the difference between first‐time breeders and experienced birds, and between males and females was significant for the Romanian population, while no difference was found between groups for the Czech population. Results on the partial selection differential on the tail length largely confirmed our former findings, although the difference between age categories for the Romanian population was less pronounced (Table 3b).

**Table 3:** General linear model testing for differences in selection for tail length across populations, sexes and age categories (first‐time breeders and experienced birds). Selection differentials (A) and gradients (B) were measured over subsequent years in Czech, CZ (2011–2017) and Romanian, RO (2012–2107) barn swallow populations. Significant effects are marked in bold

	CZ			RO			CZ + RO		
	*df*	*F*	*p*	*df*	*F*	*p*	*df*	*F*	*p*
(A) Factors influencing directional selection on tail streamers									
Age	1	1.48	.2355	**1**	**7.64**	**.0120**	1	0.12	.7330
Sex	1	1.08	.3091	**1**	**8.25**	**.0094**	1	0.29	.5910
Population	–			–			1	2.29	.1369
Age × Sex	1	2.86	.1036	1	0.22	.6417	1	2.92	.0941
Population × Age	–			–			**1**	**5.47**	**.0239**
Population × Sex	–			–			**1**	**5.04**	**.0298**
Error	24			20			45		
(B) Factors influencing directional selection on tail streamers, controlling for wing and tarsus length									
Age	1	**4.48**	**.0448**	**1**	1.71	.2063	1	0.93	.3403
Sex	1	1.09	.3079	**1**	**5.85**	**.0253**	1	0.27	.6056
Population	–			–			1	1.90	.1748
Age × Sex	1	0.27	.6090	1	0.63	.4373	1	2.92	.0941
Population × Age	–			–			**1**	**5.89**	**.0193**
Population × Sex	–			–			**1**	**4.79**	**.0339**
Error	24			20			45		

A GLM exploring the difference between sexes and age groups in the standardized directional selection differential for the brightness of the Czech barn swallows, for which we had data from multiple years, shows that the selection on this trait is similar between age categories and between males and females (sex: *F* = 0.00, *p* = .9910; age: *F* = 1.17, *p* = .2911; sex × age interaction: *F* = 0.54, *p* = .4707; *df* = 1,24). Similar results were found when we used selection gradients accounting for size (sex: *F* = 0.48, *p* = .4947; age: *F* = 1.65, *p* = .2118; sex × age interaction: *F* = 0.21, *p* = .6486; *df* = 1,24).

Regression analyses of LMEs on second‐order polynomial terms of putative secondary sexual traits showed no significant effect of the quadratic term after correction of the significance values for false discovery rate. Thus, there was no evidence for partial nonlinear selection differential on the traits that we measured (Table S1).

## 4 DISCUSSION

Divergent selection on multiple signals can be associated with decoupled developmental pathways or the maintenance of genetic constrains (Bro‐Jørgensen, 2010). Consistent with this prediction, we found that two putative secondary sexual traits, tail length and ventral coloration, in Czech and Romanian barn swallow populations were not significantly correlated, the magnitude of trait expression differed between the two populations, as did patterns of selection. In support of the prediction for the role of tail length in sexual selection in *H. r. rustica,* we found significant directional linear selection on this trait for both populations, in concordance with previous findings on other European populations (Costanzo, Ambrosini, Caprioli, Gatti, Parolini, Canova et al., 2017; Møller et al., 2006). However, the magnitude of selection on tail length differed between populations, age groups, and sexes: Selection was stronger in first‐time breeders than in experienced birds and in males than in females in the Romanian population, while differences between age groups and sexes were slight in Czech birds (Tables 2 and 3). Selection on tail length appeared to be independent of body size, because after controlling for the wing length and tarsus length, the difference between populations and groups remained the same. These results confirm previous findings, which have shown strong directional sexual selection on tail length in *H. r. rustica* males, but not females (Møller et al., 2006; Romano et al., 2017). Our findings suggest that the influence of selection may change with age and differ between closely related populations, despite a very low‐level of genomic divergence between the Czech and Romanian barn swallows (Safran et al., 2016; Wilkins et al., 2016). One possible explanation for this populational difference in selection on tail length might be related to the differences in breeding conditions. Barn swallows in the Czech population breed in small colonies of up to 40 pairs, where the competition for nest sites can be low due to the large number of empty nests built over years and because the microhabitat of breeding sites from the same barns are similar, which render the nests similarly attractive. In the Romanian population, however, barn swallows usually breed solitarily in separate stall buildings, and birds preferentially occupy nests within barns populated with farm animals (Fülöp et al., 2017), possibly because of more favorable microhabitat conditions. The difference in colonial behavior between the two populations may explain the more intense selection on tail length for early laying in first‐time breeding males from the Romanian population, where the number of favorable breeding sites is probably more limited, because experienced birds arrive first and occupy most of the preferred nest sites (PLP, pers. obs.). These differences can also be explained with the amount of variance in streamer length across age categories, because older birds are expected to be near their maximal character length and therefore there is not much variation on which selection to work. However, age groups were similar in homogeneity of variances (Levene's test: *F* < 0.02, *df* < 1,288, *p* > .9251; see Figure 1), which does not support this hypothesis.

We found that the selection on tail length is weak in experienced males and females in the Romanian, and in experienced males in the Czech population. The selection differentials for laying date on tail length fits within the range of selection previously observed on 22 European and North African barn swallow populations (Møller et al., 2006), albeit, the selection differential for experienced birds in the Romanian population is among the smallest of the studied populations of *H. r. rustica*. One explanation for the weak directional selection on tail length is related with the admixture of our populations to the proximal *H. r. transitiva*, which is characterized by a different set of sexual characters, and particularly by short tail length compared with *H. r. rustica* (Scordato & Safran, 2014; Wilkins et al., 2016). The present findings are in accordance with the evidence that substantial ongoing or recent historical gene flow is still present between *H. r. rustica* and *H. r. transitiva* (Dor et al., 2012), despite their apparent differences in morphology and life‐history traits. Disentangling factors affecting the selection on tail length of barn swallows in different populations clearly deserves further investigations.

Ventral coloration was important in mate choice for different subspecies, specifically it was shown to be under directional selection at least in the North American *H. r. erythrogaster* and in *H. r. transitiva* from the Middle East (Safran et al., 2016; Vortman et al., 2013; Wilkins et al., 2016). Our results show that ventral coloration is more intense (i.e., has lower brightness) in the Romanian than in the Czech population, and in experienced birds and males compared with first‐time breeders and females, respectively. The sexual difference in ventral coloration may suggest sexual selection on this trait, which is apparently supported by the significant directional selection of ventral coloration in first‐time breeding males on laying date. After controlling for the confounding effect of wing length and tarsus length, the selection gradient on this trait lost significance, suggesting that the advantage of dark ventral coloration in early breeding birds is determined by the correlated traits of body size. These results are consistent with what has been found in an Italian population of barn swallows (Parolini et al., 2017; Saino, Romano, Rubolini, Teplitsky, et al., 2013). In this particular Italian population, darker males had significantly higher seasonal reproductive success, but coloration did not predict the lifetime reproductive success of the birds (Costanzo, Ambrosini, Caprioli, Gatti, Parolini, Canova et al., 2017; Parolini et al., 2017). These findings show that ventral coloration may give some advantages to the bearer over the breeding season, but the underlying mechanism of this relationship is not clarified (see (Costanzo, Ambrosini, Caprioli, Gatti, Parolini, Romano et al., 2017). Future studies on different barn swallow populations of *H. r. rustica,* distributed over a gradient from the nearest *H. r. transitiva* and experiencing different environmental conditions, may clarify the role and intensity of sexual selection in generating populational phenotypic differences of multiple secondary sexual traits.

## CONFLICT OF INTEREST

None declared.

## AUTHOR'S CONTRIBUTIONS

PLP, AF, and RJS conceived the project; PLP, AF, MA, JC, RM, ANS, OT, CIV, OV, and TA collected the data; PLP and AF analyzed the data with input from RJS, MRW, OT, and TA; PLP wrote the paper with significant input from AF, MA, JC, RM, RJS, ANS, OT, CIV, OV, MRW, and TA. All authors gave final approval for publication.

## Data Availability

Selection on multiple sexual signals in two Central and Eastern European populations of the. barn swallow: Dryad https://doi.org/10.5061/dryad.64p7k2f.
