# Supplementary material for: Selection on multiple sexual signals in two Central and Eastern European populations of the barn swallow
Source: Ecol Evol. 2019 Sep 4;9(19):11277–87. doi: 10.1002/ece3.5629 (PMC6802025; doi:10.1002/ece3.5629)
Supplement: Supplementary file 1 [file ECE3-9-11277-s001.docx]

**Supporting information**

**Table S1.** Partial quadratic selection differentials (gradients, controlling for the effect of wing length and tarsus length) for the two putative sexual traits measured in first-time breeders and experienced male and female barn swallows from the Czech (CZ) and Romanian (RO) populations.

|  | CZ | | | | | RO | | | | |
| --- | --- | --- | --- | --- | --- | --- | --- | --- | --- | --- |
|  | *N* | *γ* (SE) | *t* | *P* | *P adj* | *N* | *γ* (SE) | *t* | *P* | *P adj* |
| Male |  |  |  |  |  |  |  |  |  |  |
| Tail length |  |  |  |  |  |  |  |  |  |  |
| First-time breeders | 134 | 0.003 (0.037) | 0.07 | 0.9439 | 0.9439 | 138 | 0.007 (0.036) | 0.18 | 0.8537 | 0.8537 |
| Experienced birds | 156 | 0.051 (0.053) | 0.96 | 0.3389 | 0.4983 | 159 | 0.057 (0.027) | 2.14 | 0.0341 | 0.1024 |
| Brightness |  |  |  |  |  |  |  |  |  |  |
| First-time breeders | 134 | 0.014 (0.037) | 0.39 | 0.7011 | 0.9678 | 17 | 0.215 (0.255) | 0.84 | 0.4284 | 0.6597 |
| Experienced birds | 156 | –0.007 (0.046) | –0.16 | 0.8740 | 0.8740 | 17 | –0.036 (0.083) | –0.43 | 0.6786 | 0.7564 |
| Female |  |  |  |  |  |  |  |  |  |  |
| Tail length |  |  |  |  |  |  |  |  |  |  |
| First-time breeders | 161 | 0.013 (0.030) | 0.44 | 0.6635 | 0.8211 | 178 | –0.072 (0.032) | –2.29 | 0.0230 | 0.0691 |
| Experienced birds | 150 | –0.036(0.064) | –0.57 | 0.5720 | 0.5980 | 128 | 0.017 (0.023) | 0.75 | 0.4543 | 0.6020 |
| Brightness |  |  |  |  |  |  |  |  |  |  |
| First-time breeders | 161 | –0.026 (0.029) | –0.93 | 0.3549 | 0.5323 | 14 | 0.290 (0.086) | 3.37 | 0.0280 | 0.0652 |
| Experienced birds | 150 | –0.031 (0.054) | –0.58 | 0.5658 | 0.8487 | 16 | –0.284(0.209) | –1.36 | 0.2227 | 0.6207 |
